# New genetic context of *lnu(B)* composed of two multi-resistance gene clusters in clinical *Streptococcus agalactiae* ST-19 strains

**DOI:** 10.1186/s13756-019-0563-x

**Published:** 2019-07-15

**Authors:** Kaixin Zhou, Dongan Zhu, Ying Tao, Lianyan Xie, Lizhong Han, Yibo Zhang, Jingyong Sun

**Affiliations:** 1 Department of Clinical Microbiology, Ruijin Hospital, Shanghai Jiaotong University School of Medicine, 197 Ruijin 2nd Road, Shanghai, 200025 China; 2 Department of Clinical Laboratory, Shanghai Pudong Hospital, Fudan University Pudong Medical Center, 2800 Gongwei Road, Huinan Town, Pudong, Shanghai, 201399 China; 3 Department of Hospital infection control, Ruijin Hospital, Shanghai Jiaotong University School of Medicine, 197 Ruijin 2nd Road, Shanghai, 200025 China

**Keywords:** *S. agalactiae*, *lnu(B)*, L phenotype, Composite transposon, IS1216

## Abstract

**Background:**

Clindamycin is a lincosamide antibiotic used to treat staphylococcal and streptococcal infections. Reports of clinical *Streptococcus agalactiae* isolates with the rare lincosamide resistance/macrolide susceptibility (L^R^/M^S^) phenotype are increasing worldwide. In this study, we characterised three clinical *S. agalactiae* strains with the unusual L phenotype from China.

**Methods:**

Three clinical *S. agalactiae* strains, Sag3, Sag27 and Sag4104, with the L phenotype were identified from 186 isolates collected from 2016 to 2018 in Shanghai, China. The MICs of clindamycin, erythromycin, tetracycline, levofloxacin, and penicillin were determined using Etest. PCR for the *lnu(B)* gene was conducted. Whole genome sequencing and sequence analysis were carried out to investigate the genetic context of *lnu(B)*. Efforts to transfer lincomycin resistance by conjugation and to identify the circular form by inverse PCR were made.

**Results:**

Sag3, Sag27, and Sag4104 were susceptible to erythromycin (MIC ≤0.25 mg/L) but resistant to clindamycin (MIC ≥1 mg/L). *lnu(B)* was found to be responsible for the L phenotype. *lnu(B)* in Sag3 and Sag27 were chromosomally located in an *aadE-spw-lsa(E)-lnu(B)* resistance gene cluster adjacent to an upstream 7-kb *tet(L)-cat* resistance gene cluster. Two resistance gene clusters were flanked by the IS6-like element, IS1216. Sag4104 only contained partial genes of *aadE-spw-lsa(E)-lnu(B)* resistance gene cluster and was also flanked by IS1216.

**Conclusion:**

These results established the presence of the L phenotype associated with *lnu(B)* in clinical *S. agalactiae* isolates in China. The *lnu(B)*-containing multi-resistance gene cluster possibly acts as a composite transposon flanked by IS1216 and as a vehicle for the dissemination of multidrug resistance among *S. agalactiae*.

## Background

*Streptococcus agalactiae* (group B streptococcus) is a leading cause of neonatal sepsis, meningitis, and pneumonia in many countries. It is also an important pathogen among pregnant women, immunocompromised adults, as well as the elderly [1].

The lincosamide class of antibacterial agents was first characterised in the 1960s and is now among the twenty most important antibiotic compounds [2, 3]. The most clinically relevant lincosamide, clindamycin, is frequently used to treat staphylococcal and streptococcal infections. It is also a feasible option for the treatment of β-lactamase-producing pathogens and important protozoal diseases, such as malaria [2, 4]. Clindamycin treatment has been limited by antimicrobial resistance and gastrointestinal side effects. However, it is effective for the treatment of methicillin-resistant *Staphylococcus aureus* [5, 6], especially for the empirical treatment of CA-MRSA for outpatients with skin and soft tissue infection.

Resistance to macrolides, lincosamides, and streptogramin B mediated by *erm* or *mef* is quite common in streptococci, while isolates characterized by the combination of phenotypic lincosamide resistance and macrolide susceptibility (L^R^/M^S^) are rare. Specific resistance to lincosamides is mediated by members of the *lnu* (previously *lin*) gene family, which encode nucleotidyltransferases that catalyse the adenylation of lincosamides [7, 8]. Six types of *lnu*—*lnu(A), lnu(B), lnu(C), lnu(D), lnu(E),* and *lnu(F)*—have been discovered to date [9–13]. Reports of *Streptococcus agalactiae* with the unusual L phenotype have recently increased in the United States [8], Canada [11], Spain [14], and other countries.

In this study, three clinical *S. agalactiae* isolates of the unusual L phenotype were identified. The phenotype was mediated by a nucleotidyltransferase expressed by *lnu(B)*. We investigated the genetic context of *lnu(B)* and estimated its possible formation in *S. agalactiae*. To the best of our knowledge, this is the first report on the genetic context of *lnu(B)* gene in clinical *S. agalactiae* isolates in China.

## Methods

### Bacterial strains and lincosamide resistance gene detection

Three clinical *S. agalactiae* strains with the unusual resistance phenotype (L^R^/M^S^), Sag3, Sag27 and Sag4104, were identified from 186 clinical strains collected from various sample types (including midstream urine, vaginal secretion, and blood) between 2016 to 2018 in Shanghai, China (Ruijin Hospital, School of Medicine, Shanghai Jiaotong University). All the isolates were identified using MALDI-TOF MS (bioMérieux, France), and the minimum inhibitory concentrations (MICs) were determined using VITEK2 automated systems (BioMérieux, France). Sag3 was isolated from the vaginal secretion of a pregnant female outpatient in 2016; Sag27 was isolated from perianal region of a female inpatient after bone marrow transplantation in 2018; and Sag4104 was isolated from urine sample of a female inpatient in 2018. Sag3, Sag27 and Sag4104 were investigated for the lincosamide resistance gene, *lnu(B)*, by PCR (Table 1).

**Table 1:** PCR primers used in this study

Primer	Sequence (5′–3′)	Product size (bp)
lnuB-F	ACCAAAGGAGAAGGTGACCAA	584
lnuB-R	ACCTTATCTAATCGAGCAGTGGT	
3-F	TTCGATTCCTCGTGCCTGAC	1877
3-R	AAGCGAGGTCGTAACTGGTG	
Rev-1-F	ACGCCCTGTAACGCTTGTAA	2571
Rev-1-R	TGCAAAGACCACTGCTCGAT	
Rev-2-F	GGTGAACGAAAGCCCACCTA	

### Antimicrobial susceptibility test and conjugal transfer experiments

The minimum inhibitory concentrations (MICs) of clindamycin, erythromycin, tetracycline, levofloxacin, and penicillin were determined using the Etest (bioMérieux, Marcy-l’Étoile, France). The results were interpreted based on the guidelines of the Clinical and Laboratory Standards Institute (CLSI) [15]. The transfer of lincomycin resistance was attempted from Sag3, Sag27, and Sag4104 (L^R^/M^S^) to clinical *S. agalactiae* strains (L^S^/M^R^) by the filter-mating method. Selection was performed with erythromycin (1 μg/mL) and lincomycin (1 μg/mL).

### Pulsed-field gel electrophoresis

Sag3, Sag27, and Sag4104 were digested with *Sma*I endonuclease and subjected to pulsed-field gel electrophoresis (PFGE), as described in previous studies [16, 17]. Briefly, two or three colonies were picked from a fresh plate of overnight growth and incubated in Todd-Hewitt broth for 5 h at 35 °C; this suspension was centrifuged and the pellet was re-suspended in Tris-NaCl buffer. The agarose-bacterium plugs were made and incubated overnight at 35 °C in lysis solution. The plugs were washed for several times and digested with suitable nuclease. The chromosomal digests were separated by PFGE. Pulsotypes were clustered on the basis of a cut-off of 70% similarity.

### DNA sequencing and analysis

Genomic DNA was extracted using the QIAGEN Midi Kit (Qiagen, Hilden, Germany). The DNA of Sag27 was sequenced using the PacBio RS II (Pacific Biosciences, Menlo Park, CA, USA). The reads were de novo assembled using HGAP 3.0 SMRT™ Pipe. Sag3 and Sag4104 were sequenced using the Illumina HiSeq sequencing approach. SOAP de novo (http://soap.genomics.org.cn/, v2.01) was used for genome assembly. Sequence similarity was evaluated by BLAST searches (http://www.ncbi.nlm.nih.gov). Open reading frames (ORFs) were predicted using ORF Finder (https://www.ncbi.nlm.nih.gov/orffinder/). Protein-coding genes were initially identified and annotated using RAST. Antimicrobial resistance genes were identified using ResFinder (https://cge.cbs.dtu.dk/services/ResFinder/). Multi-locus sequence typing was identified using MLST 2.0 (https://cge.cbs.dtu.dk/services/MLST/).

### Gap closure, circularisation, and integration site of the lnu(B)-carrying fragment

PCR with primers 3-F and 3-R was used to close the gap between two scaffolds around the *lnu(B)*-carrying fragment in Sag37. Outward-directed primers (Rev-1-F, Rev-1-R, Rev-2-F, and 3-R) were used to detect the circular form of the IS1216-flanked fragments (Table 1; Fig. 2).

### Nucleotide sequence accession numbers

The nucleotide sequences of Sag27/Sag4104 and the *lnu(B)*-carrying fragment of Sag3 have been deposited at DDBJ/ENA/GenBank under the accession numbers CP031556, SMRZ00000000, and MK102985, respectively.

## Results

### Characterisation of rare L^R^/M^S^*S. agalactiae* isolates

**Fig. 1:**
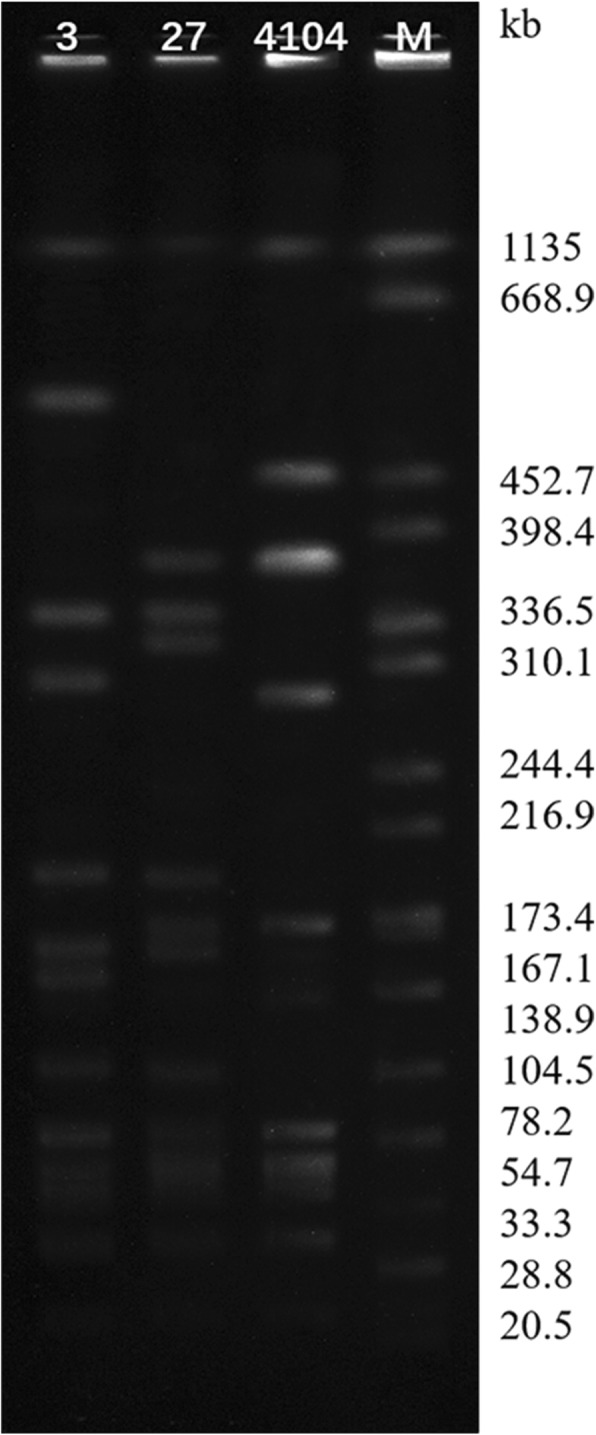
Pulsed-field gel electrophoresis (PFGE) profiles of *S. agalactiae* Sag3, Sag27, and Sag4104. PFGE analysis showed that three strains were of different pulsotypes. M, *Salmonella enterica* serotype Braenderup H9812 was digested with *Xba*I and used as a molecular size marker

The strains were susceptible (MIC ≤0.25 mg/L) to erythromycin, but resistant to clindamycin (MIC ≥1 mg/L). All the strains were classified as ST-19 and serotype III. PCR and sequencing results showed that strains contained the lincosamide resistance gene, *lnu(B)*. These results, isolation data, and resistance genes are summarised in Table 2. *Sma*I-PFGE analysis indicated that the three strains were of different pulsotypes (Fig. 1).

**Table 2:** Relevant features of the two strains Sag3 and Sag27

Strain	Year	Source	ST	Serotype	MIC (mg/L)					Resistance genes
					E	CM	TE	LEV	P	
Sag3	2016	Vaginal secretion	19	III	0.125	8	8	0.50	0.016	*lnu(B),lsa(E), aadE, cat, tet(L), tet(M)*
Sag27	2018	Perianal region	19	III	0.094	12	16	0.50	0.032	*lnu(B), aadE, cat, tet(L), tet(O)*
Sag4104	2018	urine	19	III	0.125	8	6	0.50	0.16	*lnu(B),lsa(E), tet(M)*

*E* erythromycin, *CM* clindamycin, *TE* tetracycline, *LEV* levofloxacin, *P* penicillin

### General genomic features and genetic context of lnu(B)

In the whole genome sequencing analysis, 54 total scaffolds were obtained for Sag3 with a total length of 2,292,988 bp, while 53 scaffolds were obtained for Sag4104 with a total length of 2,188,850 bp. The average G + C content of Sag3 and Sag4104 was 35.88 and 35.59%, respectively. The genome of Sag27 comprised a single circular chromosome of 2,205,229 bp in length; the average G + C content was 35.71% and a total of 2229 protein-coding sequences (CDS) were predicted.

Extensive sequence analyses revealed two *lnu(B)*-carrying fragments in both Sag3 and Sag27. In Sag 27 (CP031556, 136,248–157,861 bp), *lnu(B)* was embedded in the *aadE-spw-lsa(E)-lnu(B)* resistance gene cluster and an additional 7-kb *tet(L)-cat* resistance gene cluster was detected immediately upstream. The two clusters were flanked by the IS6-like element, IS1216. The whole fragment was 21,614 bp. A group II intron inserted into the topoisomerase I gene (*topo*) was also identified in the fragment, with high similarity to the group II intron *En.fm.*I2 first found on the *Enterococcus faecium* plasmid pVEF4 [18]. Sag4104 only contained partial genes of *aadE-spw-lsa(E)-lnu(B)* resistance gene cluster, including *lsa(E)* and *lnu(B)*. The gene cluster was also flanked by the IS6-like element, IS1216.

Comparative genome analyses identified two scaffolds in Sag3 that shared high similarity and contained all resistance genes in the abovementioned fragment in Sag27. Gap closure was performed by PCR. The final spliced fragment was 256,867 bp. Although the major drug resistance clusters were similar, there were slight differences at the head and tail regions between the two fragments. There were four additional ORFs at the tail of the fragment in Sag3 (Fig. 1). The gene cluster in Sag4104 also shared high similarity with the fragment in Sag3 and Sag27.

### Transferability

Since insertion sequences (IS) can move resistance genes as part of a composite transposon, a region bound by two copies of the same or related IS that can move as a single unit [19], several conjugal transfer experiments between Sag3/Sag27/Sag4014 and different recipient cells were performed. Even after numerous attempts, no transconjugant was obtained. This indicated that mobilisation of the *lnu(B)*-carrying fragments does not occur in *S. agalactiae*, or occurs at very low frequencies. Inverse PCR was also performed to identify the circular form of the fragments but was unsuccessful.

## Discussion

Erythromycin and clindamycin are recommended for patients who are allergic to β-lactams for the prevention or treatment of *S. agalactiae* infections [8, 20]. Erythromycin and clindamycin resistance rates in *S. agalactiae* are rising in several countries, with slight geographical variations, including the United States, Spain, and China [21–24]. Resistance to clindamycin in the absence of erythromycin resistance is relatively rare in *S. agalactiae*; however, the frequency of this phenotype has also increased in recent years [8, 14, 25, 26]. Reports of *Streptococcus agalactiae* with the unusual L phenotype have recently increased in the United States [8], Canada [11], Spain [14], and other countries. We reported three clinical *S. agalactiae* strains with the unusual L phenotype mediated by *lnu(B)* in China and further investigated the gene context of *lnu(B)*.

*lnu(B)* was chromosomally embedded in both Sag3 and Sag27 as part of a *aadE-spw-lsa(E)-lnu(B)* resistance gene cluster, while Sag4104 only contained partial genes of *aadE-spw-lsa(E)-lnu(B)* resistance gene cluster, including *lsa(E)* and *lnu(B)*. The *lnu(B)*-containing multi-resistance gene cluster in this study showed high similarity to transposable elements found in *S. agalactiae, Staphylococcus aureus, E. faecium,* and other taxa (Fig. 1), with some differences in insertion sequences in the head and tail (flanked by IS1216, IS1257, or IS1542).

Interestingly, the location of the gene cluster differs among species. The gene cluster was found on plasmids in *E. faecium* (pXD5 [27], pEF418, etc.) and was embedded either in the chromosome or on the plasmid in *S. aureus* [28]. The gene clusters were only located on the chromosomes of *S. agalactiae*. In particular, the *lnu(B)*-containing gene cluster was embedded in an integrative and conjugative element (ICE) in CUGBS591 (accession number: CP021862). The transferability of this ICE was not indicated. Coincidentally, ICESsuNC28, a mobile genetic element identified in *S. suis* harbouring the *spw-aadE-lnu(B)-lsa(E)* multidrug resistance cluster is capable of intraspecific transfer by conjugation [7]. The multidrug resistance cluster likely originated in enterococcal strains and was acquired by staphylococcal and streptococcal species [27]. Although we failed to prove that the *lnu(B)*-containing gene cluster can be transferred by mating experiments, data analysis suggested that interspecific exchange is possible. Further research is needed to characterise the mechanisms underlying its transfer.

An additional 7-kb *tet(L)-cat* resistance gene cluster was detected immediately upstream of the *lnu(B)*-containing multi-resistance gene cluster in Sag3 and Sag4104. It showed high similarity to a plasmid of *E. faecium* strain LS170308 (CP025078). Few sequences in the GenBank database show high similarity to the *tet(L)-cat* resistance gene cluster. Although the *lnu(B)*-containing multi-resistance gene cluster has been found in several species, the two gene clusters are not linked together in previously reported genomes. The tandem sequence was identified in both Sag3 and Sag27, indicating that it can be transferred as a unit among *S. agalactiae*.

With respect to the formation of this multi-drug resistance gene, the *lnu(B)*-containing and *tet(L)-cat* resistance gene clusters both originated from plasmids of enterococcal strains. Different insertion sequences (IS1216, IS257, etc.) in the plasmids formed a “translocatable unit” (TU) bound by two copies of the same IS, which can move as a single unit into plasmids or the chromosome [19]. A TU is preferentially inserted next to an existing copy of the same IS in a recipient molecule via a conserved process [19], which can explain why the *tet(L)-cat* resistance gene cluster flanked by IS1216 was detected immediately upstream of the *lnu(B)*-containing gene cluster. Thus, once a TU with a resistance gene(s) is inserted, it might become a recruiter for other TUs, resulting in the formation a multi-drug resistance gene cluster.

**Fig. 2:**
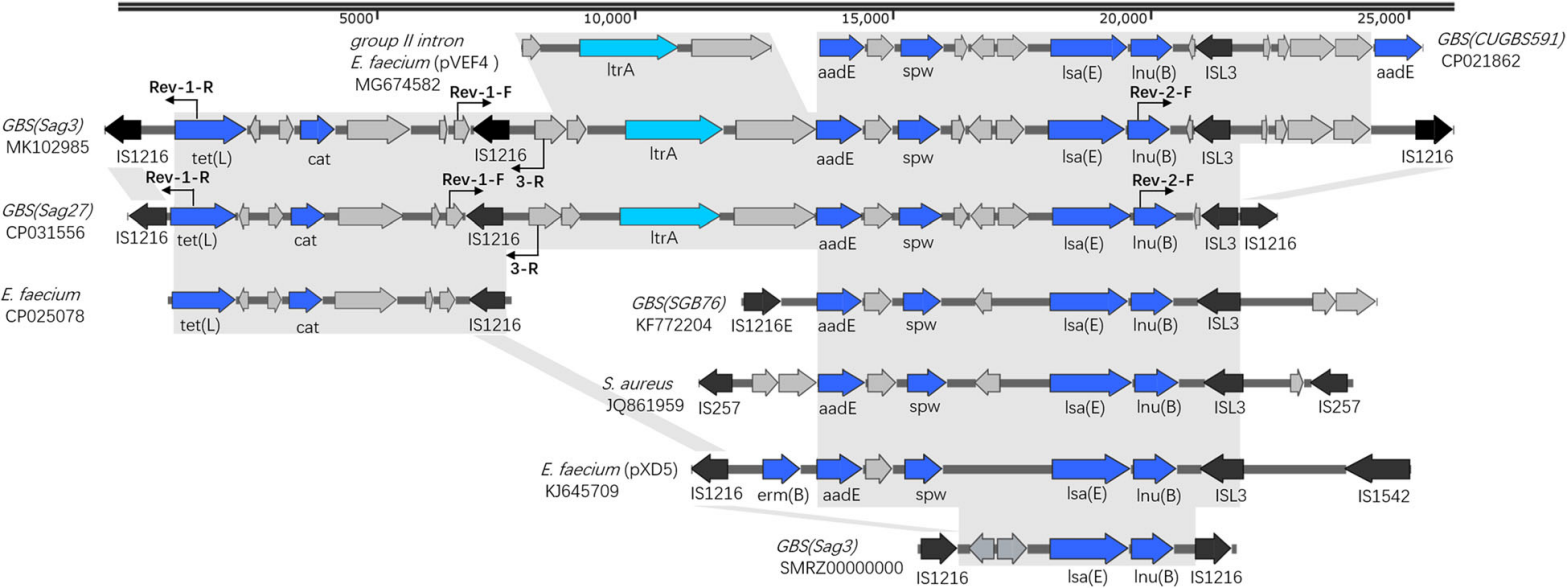
Schematic representation, drawn to scale, of the *lnu(B)*-containing multi-resistance gene clusters in Sag3/Sag27/Sag4104 and a comparison of linear DNA against the corresponding regions in different species, including *S. agalactiae*, *Staphylococcus aureus,* and *E. faecium*. The resistance genes are indicated by blue arrows and the insertion sequences are indicated by black arrows. Homologous regions are shaded in grey. Nucleotide sequence identity was at least 95% in these regions

**Fig. 3:**
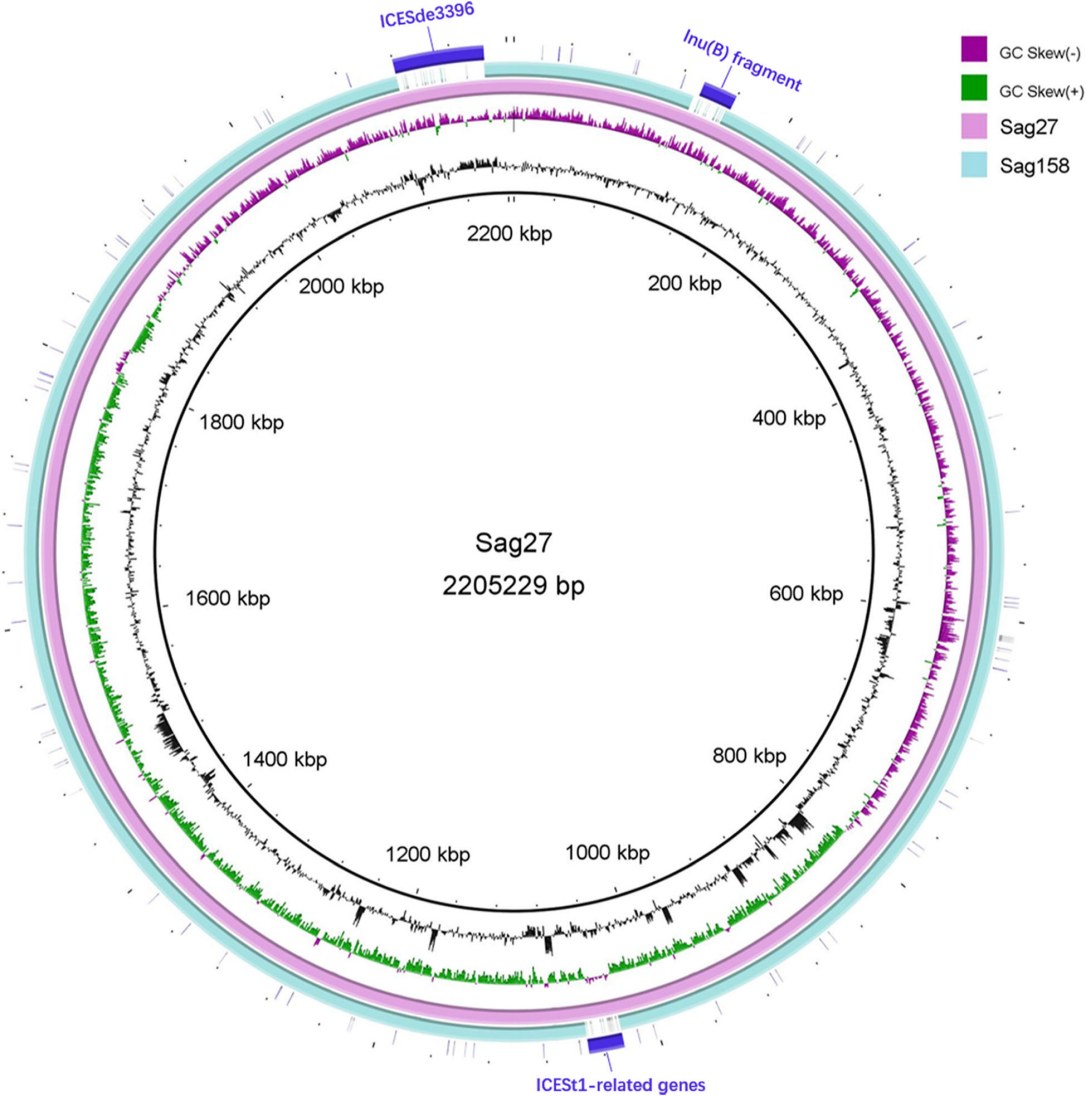
Comparative analysis of the whole genome sequences of Sag27 and Sag158 (CP019979). There are three segmental insertions in Sag27 compared to those in Sag158, including the lnu(B)-containing fragment (135–160 kb), ICESt1-related genes (1025–1050 kb), and ICESde3396 (2119–2184 kb)

Sag3, Sag27, and Sag4104 were ST-19, the most frequent ST of *S. agalactiae* in China [29]. The genome of Sag27 showed striking similarity (94%) to that of another *S. agalactiae* strain, Sag158 (accession number: CP019979), previously described by us [30] (Fig. 2). Three segmental insertions in Sag27 were detected in comparison with Sag158, including the lnu(B)-containing fragment (135–160 kb), ICESt1-related genes (1025–1050 kb), and ICESde3396 (2119–2184 kb). (Figure 3) The *lnu(B)*-containing multi-resistance gene cluster possibly acts as a composite transposon flanked by IS1216 and as a vehicle for the dissemination of multidrug resistance among ST-19 *S. agalactiae*.

## Conclusion

We reported three clinical *S. agalactiae* strains with the unusual resistance phenotype L^R^/M^S^ mediated by *lnu(B)*. Our analyses of the genetic context and transferability of *lnu(B)* provide insight into the recent spread of rare resistance phenotypes in *S. agalactiae*.

## Data Availability

The nucleotide sequences of Sag27/Sag4104 and the *lnu(B)*-carrying fragment of Sag3 have been deposited at DDBJ/ENA/GenBank under the accession numbers CP031556, SMRZ00000000, and MK102985, respectively.

## Abbreviations

CLSI: Clinical and Laboratory Standards Institute
ICE: Integrative and conjugative element
IS: Insertion sequences
MIC: The minimum inhibitory concentrations
ORF: Open reading frame
PFGE: Pulsed-field gel electrophoresis
TU: Translocatable unit
